# Therapeutic role of nifedipine in threatened preterm labor: Current evidence and future perspectives

**DOI:** 10.1002/ijgo.70816

**Published:** 2026-01-19

**Authors:** Hikaru Imatake, Yoshitsugu Chigusa, Haruta Mogami, Satoshi Morita, Masaki Mandai

**Affiliations:** ^1^ Department of Gynecology and Obstetrics, Graduate School of Medicine Kyoto University Kyoto Japan; ^2^ Department of Biomedical Statistics and Bioinformatics, Graduate School of Medicine Kyoto University Kyoto Japan

**Keywords:** calcium channel blocker, nifedipine, preterm birth, threatened preterm labor, tocolysis

## Abstract

Preterm birth occurs in approximately 10% of all pregnancies, and is not only the leading cause of neonatal mortality but also a major contributor to short‐ and long‐term morbidities due to immaturity. Preterm birth has also been linked to an increased risk of maternal cardiovascular and cerebrovascular diseases, making it a critical concern in both perinatal medicine and women's lifelong health. Effective treatment requires interventions during threatened preterm labor, and several tocolytic agents have been developed and used in clinical practice. However, no pharmacological agent has been shown to prolong gestation and improve neonatal outcomes. Nifedipine, a calcium channel blocker, is widely used as a first‐line tocolytic agent because of its oral administration route and relatively favorable safety profile compared with other drugs. Evidence from randomized controlled trials, meta‐analyses, and Cochrane reviews suggests that nifedipine can delay delivery for a short period; however, robust evidence demonstrating sustained prolongation of pregnancy or improved neonatal survival is still lacking. Moreover, data on maternal hemodynamic changes and fetal effects are limited, highlighting the need for optimal dosing strategies and monitoring protocols. In this study, we discuss the clinical significance and limitations of nifedipine in the management of threatened preterm labor and outlined future directions. Future studies should involve large and homogeneous populations, continuous assessment of maternal hemodynamics, and application of novel biomarkers to support individualized therapy. Accumulation of such evidence is expected to optimize the management of threatened preterm labor and ultimately improve outcomes for mothers and infants.

## 1 INTRODUCTION

Human pregnancy typically lasts for approximately 280 days or 40 weeks, from the first day of the last menstrual period. Deliveries occurring between 37 and <42 weeks of gestation are defined as term births, whereas those occurring before 37 weeks are classified as preterm births. Preterm birth is not only the leading cause of neonatal mortality but also a major contributor to short‐ and long‐term morbidities, including respiratory distress syndrome, chronic lung disease, intraventricular hemorrhage, sepsis, cerebral palsy, and visual and hearing impairments.^1^ Moreover, preterm birth has been associated with an increased risk of subsequent maternal cardiovascular and cerebrovascular disease.^2^, ^3^ Thus, preterm birth represents a major concern not only in perinatal medicine but also in women's lifelong and public health.

Globally, the incidence of preterm birth remains high, which is estimated at approximately 9.9%. In Japan, the rate is 5.7%, which is far from negligible.^4^ Preterm birth can be broadly classified into two categories: spontaneous preterm birth, in which uterine contractions and cervical dilation occur before 37 weeks of gestation, leading to delivery, and iatrogenic preterm birth, in which pregnancy must be terminated for maternal or fetal indications, such as hypertensive disorders of pregnancy or fetal compromise. The relative contribution of these categories varies by country, but in developed nations, such as the UK, the United States, and Japan, approximately half of preterm births are thought to be spontaneous.^5^, ^6^ In iatrogenic preterm births, the underlying conditions are diverse and are often not directly addressed by preterm birth interventions, making prevention particularly challenging. In contrast, even partial success in managing spontaneous preterm labor is likely to reduce neonatal mortality and morbidity. Further, prolonged gestation in cases of threatened preterm labor, even if preterm delivery cannot be entirely prevented, allows time for crucial interventions, such as antenatal corticosteroid administration to enhance fetal lung maturity, maternal transfer to facilities with neonatal intensive care units, and magnesium sulfate therapy for fetal neuroprotection.^7^ Therefore, the effective treatment of preterm labor represents one of the most urgent and important challenges in current perinatal medicine.

In this review, we focus on nifedipine, a calcium channel blocker originally developed as an antihypertensive agent but now widely used as an oral tocolytic, particularly in Europe and North America, because of its ease of administration and relatively favorable maternal safety profile. Despite its widespread use, the evidence regarding its efficacy in threatened preterm labor remains inconsistent across clinical studies. Reported benefits in prolonging pregnancy are modest, and improvements in neonatal outcomes have not been clearly demonstrated. This review summarizes the existing evidence and discusses the pharmacological properties, efficacy, and safety of nifedipine in the management of threatened preterm labor.

## 2 HISTORY AND CURRENT STATUS OF PRETERM BIRTH TREATMENT

The primary goal of preterm birth management is to suppress uterine contractions in the setting of threatened preterm labor, thereby preventing progression to delivery, with the aim of prolonging gestation. In other words, the treatment of preterm birth is synonymous with that of threatened preterm labor. Although the ideal outcome is to maintain pregnancy until 37 weeks of gestation or beyond, even a modest prolongation of pregnancy before this point can significantly improve neonatal outcomes; therefore, this is regarded as a clinically meaningful therapeutic achievement. Several tocolytic agents have been developed and used clinically over the past 70 years. Relaxin, first reported in 1955, was the first of such attempts.^8^ Subsequently, in 1959, magnesium sulfate was reported to extend gestation; however, later studies concluded that it was insufficient in preventing preterm birth.^9^ Nevertheless, magnesium sulfate has been shown to exert neuroprotective effects in preterm infants, and it continues to be administered to mothers with threatened preterm labor for this specific purpose.^7^

From 1961 onward, β‐adrenergic agonists were introduced as therapeutic agents for threatened preterm labor.^10^ In 1980, the U.S. Food and Drug Administration approved ritodrine hydrochloride as a tocolytic agent, which was widely used until the 2000s. Meta‐analyses demonstrated that ritodrine significantly reduced the incidence of delivery within 48 h but did not reduce the overall rate of preterm birth or improve neonatal outcomes.^11^ Moreover, owing to serious maternal cardiovascular side effects, the use of ritodrine has been restricted worldwide to short‐term intravenous administration, which is typically limited to 48 h. An exception is Japan, where long‐term administration is still practiced in some cases. Around the same period, prostaglandin synthesis inhibitors were introduced for the management of threatened preterm labor, owing to their potent inhibitory effects on uterine contractions. However, their use has been limited by severe adverse fetal effects, such as premature closure of the ductus arteriosus and oligohydramnios, and the lack of clear evidence of improved neonatal outcomes compared with placebo.^12^ As a result, these agents are generally not recommended for routine treatment of preterm labor.

In 1978, Ulmsten et al. showed that nifedipine, a calcium channel blocker, inhibited uterine contractions in non‐pregnant women,^13^ and in 1980 the first clinical report of nifedipine for threatened preterm labor was published.^14^ Since then, despite ongoing scrutiny and debate, nifedipine has become widely accepted as a first‐line tocolytic agent in Europe and the United States.^15^ In the UK, the National Institute for Health and Care Excellence guidelines classify nifedipine use for threatened preterm labor as off‐label; nevertheless, they recommend that nifedipine should be “considered” for cases between 24 + 0 and 25 + 6 weeks of gestation and “offered” for cases between 26 + 0 and 33 + 6 weeks, thereby establishing nifedipine as the practical first choice tocolytic.^16^ In the United States, the American College of Obstetricians and Gynecologists practice bulletin does not endorse a specific tocolytic agent; rather, magnesium sulfate is administered for fetal neuroprotection in threatened preterm labor, and nifedipine might be used cautiously if uterine contractions persist.^17^ However, the World Association of Perinatal Medicine guidelines explicitly recommend nifedipine as the first‐line agent for the treatment of threatened preterm labor up to 33 + 6 weeks of gestation.^18^ In Japan, although nifedipine is not covered by the national health insurance system and is considered for off‐label use, the national obstetric practice guidelines acknowledge its potential benefits and risks, stating that its administration might be considered after obtaining informed consent from patients.^19^ Additionally, oxytocin receptor antagonists, such as atosiban, were introduced in 1994. However, the most recent randomized controlled trials demonstrated that although atosiban prolongs pregnancy beyond 48 h compared with placebo, it does not significantly delay delivery overall, and it fails to improve the primary endpoint of neonatal outcomes.^20^

## 3 PHARMACOLOGICAL PROPERTIES OF NIFEDIPINE

Calcium channel blockers were first introduced in 1967 when Fleckenstein, in the course of a study on anti‐angiogenic agents, designated verapamil as a “calcium antagonist” based on its efficacy as a coronary vasodilator.^21^ Nifedipine, developed in 1971, soon entered clinical use for the treatment of angina pectoris. Initially, nifedipine was recognized to have a pronounced antihypertensive effect, and it naturally became a major antihypertensive agent. Calcium channel blockers are currently among the most prescribed antihypertensive drugs worldwide.^22^ In Japan, the national guidelines for hypertension management list angiotensin II receptor blockers, angiotensin‐converting enzyme inhibitors, and diuretics as first‐line pharmacologic therapy.^23^

Calcium channel blockers exert their effects by binding to voltage‐dependent calcium channels on the cell membrane and inhibiting calcium influx. This leads to peripheral vasodilation, with a consequent reduction in blood pressure, coronary vasodilation, negative inotropy, and suppression of atrioventricular conduction. The L‐type calcium channels targeted by nifedipine are not only expressed in cardiac and vascular smooth muscles but also in non‐vascular smooth muscles, such as the uterus and gastrointestinal tract, as well as in non‐contractile tissues, including the pancreas and adrenal glands.^24^

Nifedipine is available in three formulations: immediate‐, slow‐, and controlled‐release preparations. Previously, sublingual administration of immediate‐release nifedipine was occasionally used to achieve rapid onset; however, this practice is no longer recommended because of the risk of excessive hypotension leading to reflex tachycardia, cerebral ischemia, or myocardial ischemia.^23^ In the context of threatened preterm labor, there is no consensus regarding the optimal formulation, dosage, or dosing interval, and various treatment protocols have been reported. Table 1 summarizes the administration methods of nifedipine used in clinical studies. Oral administration of either immediate‐ or slow‐release nifedipine at a dose of 20 mg every 4–8 h is common.^25^, ^26^, ^27^, ^28^, ^29^, ^30^ Other regimens have included sublingual administration of immediate‐release formulations,^31^ once‐daily oral administration of 30 mg controlled‐release tablets,^32^ or repeated oral doses of 20 mg immediate‐release nifedipine every 30–90 min until uterine contractions are controlled.^28^, ^29^ As outlined in UpToDate, a widely cited approach is to initiate therapy with 20–30 mg of immediate‐release nifedipine orally, followed by 10–20 mg every 3–8 h as needed.^33^

**TABLE 1:** Nifedipine dosing regimens for tocolysis in threatened preterm labor.

References	Loading dose			Maintenance dose		
	Formulation	Single dose	Dosing interval	Formulation	Single dose	Dosing interval
Songthamwat et al. (2018)^28^	IR	20 mg	30 min Until uterine contractions improve	IR	20 mg	8 h
Hawkins et al. (2021)^29^	IR	20 mg	90 min Until uterine contractions improve	IR	20 mg	4 h
Leal‐Junior et al. (2020)^31^	IR (oral/sublingual)	20 mg	30 min Until uterine contractions improve	IR	20 mg	Within 24 h: 6 h 24–48 h: 8 h
Lyell et al. (2008)^25^ Roos et al. (2013)^26^ Vis et al. (2015)^27^	None			SR	20 mg	6 h
Parry et al. (2014)^30^	None			SR	20 mg	8 h
Danti et al. (2014)^32^	None			CR	30 mg	24 h
Our Institute	None			CR	20 mg	8–12 h

Abbreviations: CR, controlled release; IR, immediate release; SR, slow release.

Although pharmacokinetic data for nifedipine in pregnant women are limited, the key parameters relevant to obstetric use can be derived from data obtained in non‐pregnant healthy adults. Table 2 summarizes the onset of action (Tmax), elimination half‐life (T1/2), peak plasma concentration (Cmax), systemic exposure (area under the curve), and fetal‐to‐maternal (F/M) concentration ratio after single oral administration of immediate‐, slow‐, and controlled‐release formulations.^34^, ^35^, ^36^ Nifedipine has a relatively small molecular weight (366 Da) and is known to cross the placenta, with reported F/M concentration ratios ranging from 0.5 to 0.9. Importantly, a prospective cohort study involving 299 pregnancies exposed to calcium channel blockers during the first trimester (including 76 exposed to nifedipine) found no increased risk of major congenital malformations compared with unexposed controls.^37^ Consistent with these findings, several other studies have likewise demonstrated no association between nifedipine exposure in early pregnancy and an elevated risk of congenital anomalies.^38^, ^39^

**TABLE 2:** Pharmacokinetics of nifedipine (single oral administration).

	Tmax (h)^a^	T1/2 (h)^a^	Cmax (ng/ml)^a^	AUC (ng/mL/h)^a^	F/M ratio
Immediate‐release 10 mg	0.6 ± 0.1	3.4 ± 0.4	73.5 ± 17.5	125.0 ± 178.2	0.76–0.93
Slow‐release 20 mg	2.8 ± 0.6	3.6 ± 1.6	70.7 ± 19.9	298 ± 137	0.53
Controlled‐release 20 mg	6.4 ± 4.9	7.1 ± 2.8	28.0 ± 14.2	419 ± 259	0.77

Abbreviations: AUC, area under the blood concentration time curve; Cmax, maximum drug concentration; F/M ratio, fetal maternal serum ratio; Tmax, maximum drug concentration time; T1/2, half‐life period.

^a^ Measured in non‐pregnant, healthy individuals.

The adverse effects of nifedipine are largely attributable to its vasodilatory action and include hypotension, palpitations, and headache, with reflex tachycardia occasionally occurring secondary to decreased blood pressure.^40^ Gingival hyperplasia, although rare, has also been reported.^41^ In threatened preterm labor, headaches are frequently observed following drug administration; however, severe maternal hypotension or tachycardia requiring hemodynamic resuscitation is uncommon. Nevertheless, van Veen et al. described a case at 26 + 5 weeks of gestation in which marked maternal hypotension developed after nifedipine administration, followed by profound fetal bradycardia and ultimately intrauterine fetal demise.^42^ To date, this is considered the most severe adverse event associated with nifedipine treatment. Importantly, in this case, the drug was administered by chewing an immediate‐release tablet, followed by an additional dose 15 min later, which resulted in a rapid surge in plasma concentration. Moreover, despite the short half‐life of nifedipine (approximately 1.3 h), hypotension persisted for 6 h, placental examination revealed approximately 20% infarction, and no fetal autopsy was performed. These findings have led researchers to question the direct causal relationship between nifedipine administration and adverse outcomes.^43^

Nevertheless, the use of nifedipine for threatened preterm labor involves the administration of an antihypertensive agent to normotensive pregnant women, and robust evidence regarding the maternal and fetal effect of its adverse effects remains limited. To date, only one prospective and one retrospective observational study have specifically assessed changes in blood pressure and heart rate following nifedipine administration.^44^, ^45^ In a prospective study by Luewan et al. involving 157 women, a protocol of four doses of 10 mg immediate‐release nifedipine at 15‐min intervals, followed by 60 mg of controlled‐release nifedipine 4–6 h later, was employed. At 60 min after initiation, both systolic and diastolic blood pressures decreased by an average of approximately 4 mmHg, and hypotension (defined as a ≥ 15 mmHg decline) was observed in approximately 17% of participants.^44^ In a retrospective analysis of 138 patients by Yamasato et al., blood pressure decreased by an average of 5 mmHg at approximately 8 h after administration, but hypotension incidence (systolic <90 mmHg or diastolic <60 mmHg) did not increase.^45^ Tachycardia (heart rate ≥100 bpm) occurred more frequently, although mean increase was modest at 4 bpm.^45^ Importantly, both studies used repeated dosing of immediate‐release nifedipine, which differed substantially from once‐ or twice‐daily administration of slow‐ or controlled‐release formulations. Against this background, in our yet‐to‐be‐published analysis, we observed that the administration of controlled‐release nifedipine (median dose, 40 mg/day) to normotensive pregnant women with threatened preterm labor resulted in an average reduction of approximately 7 mmHg in systolic and diastolic blood pressures by the following day. Hypotension, defined as a ≥15 mmHg decline, was noted in 42.7% for systolic and 34.3% for diastolic blood pressure. These findings underscore the importance of careful blood pressure monitoring after nifedipine therapy initiation, even in normotensive women with threatened preterm labor.

## 4 EFFICACY OF NIFEDIPINE FOR THREATENED PRETERM LABOR

We searched the literature using PubMed on April 1, 2025, with the keywords “nifedipine” AND (“preterm labor” OR “tocolysis”). We primarily included randomized controlled trials using placebo as a comparator and restricted the search to English‐language human studies. Although these criteria captured the majority of relevant articles, we additionally reviewed randomized trials cited in the latest Cochrane Review on tocolytics and included eligible studies that were not retrieved in the initial PubMed search. Reference lists of key articles were also screened to identify further relevant trials. Case reports, observational studies, reviews, and non‐English publications were excluded.

Several randomized controlled trials have evaluated the efficacy of nifedipine in the management of threatened preterm labor. However, these studies differed substantially in terms of patient population, treatment protocols, and primary endpoints. Primary outcomes were broadly categorized into two domains: prolonged pregnancy and neonatal outcomes.

Ara et al. conducted a randomized placebo‐controlled trial in women with threatened preterm labor at 30–34 weeks of gestation using prolonged pregnancy beyond 48 h after treatment initiation as primary endpoint.^46^ They reported that prolongation of pregnancy for at least 48 h was achieved in 77.8% (35/45) of women in a nifedipine group compared with 11.4% (5/44) in a placebo group, representing a significant benefit of nifedipine.^46^ In contrast, Songthamwat et al. performed a randomized, double‐blind, placebo‐controlled trial among women at 24–34 weeks of gestation who presented with painful, regular uterine contractions occurring at least once every 10 min, with the same primary outcome.^28^ At 48 h after treatment initiation, uterine contraction arrest was observed in 77.6% (80/103) of the nifedipine group versus 49.5% (51/103) of the placebo group (adjusted relative risk [aRR] 1.52, 95% confidence interval [CI]: 1.22–1.91), demonstrating a tocolytic effect of nifedipine.^28^ However, pregnancy continuation beyond 48 h was comparable between groups (90.3% [93/103] with nifedipine and 87.4% [90/103] with placebo [aRR, 1.05; 95% CI: 0.95–1.15]) indicating no clear advantage in pregnancy prolongation.^28^ Further, Lyell et al. conducted a randomized, double‐blind, placebo‐controlled trial in women aged 24–34 weeks whose uterine contractions with cervical dilation were initially controlled using either magnesium sulfate or oral nifedipine.^25^ In this study, the primary endpoint was pregnancy prolongation to at least 37 weeks. The results showed no significant difference: 39% of women in a nifedipine group and 37% in a placebo group achieved delivery at 37 weeks or later.^25^ Similarly, no difference was observed between the groups regarding maintenance of pregnancy at 48 h after treatment initiation.^25^

The APOSTEL‐I trial was a randomized, double‐blind, placebo‐controlled trial of women with threatened preterm labor at 24–34 weeks of gestation who tested negative for fetal fibronectin, with delivery within 7 days as primary endpoint.^27^ Results showed that delivery within 7 days occurred in 8.1% (3/37) of a nifedipine group and 2.8% (1/36) of a placebo group.^27^ Median gestational age at delivery was 37 + 0 weeks in the nifedipine group versus 38 + 2 weeks in the placebo group, suggesting a longer gestational duration in the placebo group.^27^ However, it should be noted that the trial enrolled only 73 participants compared with a planned 220 participants, and there was an imbalance in baseline characteristics, with a higher proportion of twin pregnancies and women with a history of preterm birth in the nifedipine group. In another randomized, double‐blind, placebo‐controlled trial of women at 24–32 weeks of gestation with cervical shortening but without uterine contractions, prophylactic nifedipine was evaluated, with preterm birth before 37 weeks as the primary outcome.^32^ The rate of preterm birth was 11.4% (5/44) in a nifedipine group compared with 19.0% (8/42) in a placebo group, a difference that did not reach statistical significance but suggested a trend toward reduced preterm birth with nifedipine.^32^ Notably, a stratified analysis limited to multiparous women demonstrated a significant reduction in the number of preterm births in the nifedipine group. Other randomized, double‐blind, placebo‐controlled trials have also investigated outcomes such as prolongation of pregnancy for at least 7 days or preterm birth before 37 weeks; however, none have provided definitive evidence supporting nifedipine efficacy.^29^, ^30^

Two randomized, double‐blind, placebo‐controlled trials have specifically examined neonatal outcomes following nifedipine therapy for threatened preterm labor: the APOSTEL‐II and APOSTEL‐IV trials.^26^, ^47^ The APOSTEL‐II trial enrolled women at 26–32 weeks of gestation with threatened preterm labor who remained undelivered for at least 48 h after treatment initiation and completed a full course of antenatal corticosteroids.^26^ The participants were then randomized to receive maintenance nifedipine or placebo, with neonatal morbidity as the primary outcome.^26^ The incidence of neonatal complications was 11.9% (24/201) in a nifedipine group and 13.7% (28/205) in a placebo group (RR, 0.78; 95% CI: 0.53–1.45), showing no significant difference. Median gestational age at delivery was approximately 34 weeks in both groups.^26^ A follow‐up developmental assessment at 2 years of age revealed conflicting findings: fine motor problems were significantly more frequent in the nifedipine group (22.2% vs. 7.6%, odds ratio [OR], 3.43; 95% CI: 1.29–9.14, *P* = 0.01), whereas deficits in problem‐solving ability were significantly less frequent (21.1% vs. 29.1%; OR, 0.27; 95% CI: 0.08–0.95, *P* = 0.04).^48^ No overall differences were observed in the prevalence of global developmental delay.^48^ The APOSTEL‐IV trial investigated women with preterm pre‐labor rupture of membranes at 24–34 weeks of gestation in the absence of uterine contractions.^47^ Neonatal complications occurred in 33.3% (9/27) of infants in the nifedipine group and 32.1% (9/28) in the placebo group (RR, 1.04; 95% CI: 0.49–2.20), with no significant difference.^47^ Median gestational age at delivery was 29.9 weeks in a nifedipine group versus 27.0 weeks in a placebo group.^47^ However, the trial was terminated early because of slow recruitment, with only 50 participants enrolled out of the planned 120. This limitation must be considered when interpreting the results.

In summary, previous studies evaluating the efficacy of nifedipine for threatened preterm labor have yielded inconsistent findings, largely due to differences in study populations and outcome measures. A 2022 Cochrane Review incorporating these trials through a network meta‐analysis concluded that nifedipine, compared with placebo or no treatment, significantly delayed delivery within 48 h (RR, 1.16; 95% CI: 1.07–1.24) and might also reduce deliveries within 7 days of treatment initiation (RR, 1.15; 95% CI: 1.04–1.27).^11^ However, nifedipine did not significantly reduce preterm birth before 37 weeks of gestation (RR, 0.91; 95% CI: 0.78–1.07), and mean prolongation of pregnancy relative to placebo was limited to 4.66 days (95% CI: 0.13–9.19).^11^ With respect to neonatal outcomes, nifedipine was associated with reductions in low birth weight (<2500 g; RR, 0.80; 95% CI: 0.69–0.93), neurodevelopmental impairment (RR, 0.51; 95% CI: 0.30–0.85), and respiratory distress (RR, 0.68; 95% CI: 0.53–0.88).^11^ In contrast, no significant effects were observed for neonatal death within 28 days (RR, 0.84; 95% CI: 0.44–1.57), gastrointestinal complications (RR, 0.57; 95% CI: 0.23–1.41), or neonatal infection (RR, 0.79; 95% CI: 0.53–1.17).^11^ Additionally, meta‐analyses comparing nifedipine with nitroglycerin, ritodrine hydrochloride, and magnesium sulfate demonstrated that nifedipine was superior in prolonging pregnancy within 48 h, although its benefits beyond this period appeared to be limited.^49^

## 5 DISCUSSION

The therapeutic efficacy of nifedipine for threatened preterm labor has been evaluated by multiple research groups, and several randomized controlled trials as well as meta‐analyses have been reported to date. This represents a distinguishing feature compared with other tocolytic agents, indicating that nifedipine is supported by a relatively substantial body of clinical evidence. Indeed, a modest but reproducible effect on short‐term prolongation of pregnancy has been consistently demonstrated. However, as summarized in Table 3, considerable heterogeneity exists among these studies with respect to gestational age at enrollment, definitions of threatened preterm labor, and criteria for treatment initiation. In addition, primary endpoints have not been uniformly defined across trials. Such heterogeneity complicates direct comparisons between studies and contributes to variability in the interpretation of their results. Moreover, although the efficacy of tocolytic therapy should ultimately be assessed based on improvements in neonatal outcomes, relatively few studies have incorporated neonatal outcomes as primary endpoints. This might, in part, reflect the difficulty of detecting clinically meaningful differences in neonatal outcomes when pregnancy is prolonged by only a few days, which has likely limited the adoption of such study designs.

**TABLE 3:** Comparative summary of randomized controlled trials evaluating nifedipine for threatened preterm labor.

References	GA at randomization	Prolongation of pregnancy for 48 h	Prolongation of pregnancy for 7 days	Preterm delivery	GA at delivery (week)	Prolongation of pregnancy (days)	Adverse perinatal outcome
Ara et al. (2008)^46^	30–34	**35/45 (77.8)** **vs. 5/44 (11.3)** ^**a**^ ***P* < 0.05**	–	–	–	–	–
Songthamwat et al. (2018)^28^	24–36	**93/103 (90.3)** **vs. 90/103 (87.4)** **RR 1.03 (0.95–1.15)**	–	26/99 (26.3) vs. 26/103 (25.2)	37.26 ± 1.56 vs. 37.17 ± 1.84 *P =* 0.71	3.6 ± 2.7 vs. 3.7 ± 2.9 *P =* 0.70	Neonatal outcomes were similar
Lyell et al. (2008)^25^	24–34	33/33 (100) vs. 33/35 (94.3) *P =* 0.49	31/33 (94) vs. 31/35 (89) *P =* 0.67	**20/33 (60.6) vs. 22/35 (62.9)**	35.0 ± 3.4 vs. 35.2 ± 3.5 *P =* 0.82	33.5 ± 19.9 vs. 32.6 ± 21.4 *P =* 0.81	16/48 (33.3) vs. 10/43 (23.3) *P =* 0.36
Vis et al. (2015)^27^	24–34	–	**34/37 (91.9)** **vs. 35/36 (97.2)**	18/37 (48.6) vs. 8/36 (22.2) ARR −26 (−8.0)	37.0 (34.9–38.7) vs. 38.3 (37.0–39.9) *P =* 0.008	‐	3/37 (8.1) vs. 0/36 (0) ARR −8.1 (0.38)
Danti et al. (2014)^32^	24–32	–	–	**8/42 (19.0)** **vs. 5/44 (11.4)** ***P =* 0.32**	38.3 ± 2.5 vs. 38.5 ± 1.8 *P =* 0.67	74.9 ± 23.6 vs. 77.3 ± 24.5 *P =* 0.65	Neonatal outcomes were similar
Hawkins et al. (2021)^29^	28–33	36/46 (78) vs. 30/42 (71) RR 1.1 (0.9–1.4)	33/46 (72) vs. 29/42 (69) RR 1.0 (0.8–1.4)	**24/46 (52)** **vs. 20/42 (48)** **RR 1.1 (0.7–1.7)**	36.0 ± 3.0 vs. 35.7 ± 3.4 MD 0.3 (−1.1–1.7)	36.7 (2.6–47.9) vs. 31.0 (1.3–43.7) MD 5.7 (−2.4–13.9)	Neonatal outcomes were similar
Parry et al. (2014)^30^	24–34	–	**22/29 (75.9)** **vs. 25/31 (80.6)** **RR 0.94 (0.72–1.23)**	12/29 (41.4) vs. 12/31 (38.7) RR 1.1 (0.58–2.0)	36.1 ± 5.1 vs. 36.8 ± 3.6 *P =* 0.027	**46.8 ± 33.3** **vs. 50.8 ± 29.2** ***P =* 0.48**	**–**
Roos et al. (2013)^26^	26–32	–	–	137/201 (68.2) vs. 137/205 (66.8) RR 1.0 (0.89–1.2)	34.1 ± 4.0 vs. 34.2 ± 4.0 HR 1.0 (0.83–1.2)	30 (7–56) vs. 32 (4–59) HR 1.0 (0.84–1.2)	**24/201 (11.9)** **vs. 28/205 (13.7)** **RR 0.87 (0.53–1.45)**
Nijman et al. (2016)^47^	24–33	23/25 (92.0) vs. 25/25 (100) RR 0.92 (0.92–1.05)	16/25 (64.0) vs. 15/25 (60.0) *P =* 0.50	–	32.0 (29.1–33.3) vs. 30.0 (26.3–32.1) *P =* 0.115	11 (4–19) vs. 8 (5–25) HR 1.02 (0.58–1.8)	**9/27 (33.3)** **vs. 9/28 (32.1)** **RR 1.04 (0.43–2.5)**

*Note*: Data are nifedipine versus placebo, *n*/*N* (%), mean ± standard deviation or median (25th percentile–75th percentile). RR, MD, and HR are presented with 95% confidence intervals, and ARR is presented with 95% one‐sided confidence limit. *P*‐values are shown only when reported in the original studies. Bold text indicates primary endpoints.

Abbreviations: ARR, absolute risk reduction; GA, gestational age; HR, hazard ratio; MD, mean difference; RR, relative risk.

^a^ Delivery between 48 hours and 7 days.

In the Cochrane Review, nifedipine was associated with a mean prolongation of pregnancy of 4.66 days compared with placebo^11^; however, the clinical significance of this finding has not yet been conclusively established. Nonetheless, a prolongation of pregnancy by approximately 4.66 days might provide a critical window to complete antenatal corticosteroid administration, initiate magnesium sulfate for fetal neuroprotection, and arrange maternal transfer to a tertiary care center. Although robust evidence demonstrating that these interventions translate into improved neonatal outcomes in this specific context remains limited, the ability to secure adequate time for optimal perinatal management might itself carry clinical value. Moreover, previous study has shown that even a 1‐week increase in gestational age is associated with improved neonatal survival and reduced morbidity,^50^ suggesting that a pregnancy prolongation of only a few days might still have the potential to favorably influence neonatal outcomes.

Taken together, several important research gaps remain. First, it remains unclear which patient characteristics or clinical contexts are associated with the greatest therapeutic benefit from nifedipine. Second, definitive evidence is lacking as to whether pregnancy prolongation achieved with nifedipine translates into improvements in short‐ or long‐term neonatal outcomes. Third, data quantifying the incidence and severity of maternal adverse effects, particularly hypotension and other hemodynamic changes, as well as potential fetal effects, remain limited. Fourth, optimal treatment regimens, including formulation selection and dosing strategies, have yet to be established. To address these gaps, future studies should focus on larger and more homogeneous populations, with prospective designs that incorporate short‐ and long‐term neonatal outcomes as primary endpoints, to more precisely define the clinical efficacy of nifedipine. In addition, given that nifedipine is administered to normotensive pregnant women despite its antihypertensive properties, systematic evaluation of maternal hemodynamic responses and fetal effects is warranted to better characterize its safety profile. Further, integrated investigations examining optimal formulation choice, dosing strategies, and monitoring approaches will be essential to maximize therapeutic benefit while minimizing potential risks.

## 6 CONCLUSION

Nifedipine has been used for many years in the management of threatened preterm labor as an orally administered tocolytic with a relatively favorable tolerability profile. In this review, we have summarized the available clinical evidence and outlined the pharmacological properties, efficacy, and safety of nifedipine based on published studies. Existing evidence suggests that nifedipine provides a modest but reproducible short‐term prolongation of pregnancy; however, its precise clinical role warrants cautious interpretation. At present, no alternative tocolytic agent has demonstrated clearly superior efficacy with a more favorable maternal safety profile, and nifedipine is therefore likely to remain an important therapeutic option in clinical practice for the foreseeable future. Further accumulation of high‐quality evidence is expected to clarify the appropriate role of nifedipine in the management of threatened preterm labor.

## AUTHOR CONTRIBUTIONS

Y.C. and H.I. conceived the concept of this review and drafted the manuscript. S.M. contributed to the interpretation of relevant literature and provided intellectual input. H.M. critically revised and refined the manuscript for important intellectual content. M.M. supervised the entire process. All authors read and approved the final version of the manuscript and agree to be accountable for all aspects of the work.

## FUNDING INFORMATION

This study was supported by Division of Graduate Studies SPRING Program from Division of Graduate Studies in Kyoto University.

## CONFLICT OF INTEREST STATEMENT

The authors declare that they have no conflicts of interest.

## Data Availability

Data sharing is not applicable to this article as no new data were created or analyzed in this study.

## References

[1] Perin J , Mulick A , Yeung D , et al. Global, regional, and national causes of under‐5 mortality in 2000–19: an updated systematic analysis with implications for the sustainable development goals. Lancet Child Adolesc Health. 2022;6(2):106‐115. 34800370 10.1016/S2352-4642(21)00311-4 PMC8786667

[2] Wu P , Gulati M , Kwok CS , et al. Preterm delivery and future risk of maternal cardiovascular disease: a systematic review and meta‐analysis. J Am Heart Assoc. 2018;7(2): e007809. 29335319 10.1161/JAHA.117.007809 PMC5850169

[3] Crump C , Sundquist J , Sundquist K . Preterm delivery and long‐term risk of stroke in women: a National Cohort and Cosibling study. Circulation. 2021;143(21):2032‐2044. 33966449 10.1161/CIRCULATIONAHA.120.052268 PMC8154738

[4] Ohuma EO , Moller AB , Bradley E , et al. National, regional, and global estimates of preterm birth in 2020, with trends from 2010: a systematic analysis. Lancet. 2023;402(10409):1261‐1271. 37805217 10.1016/S0140-6736(23)00878-4

[5] Gyamfi‐Bannerman C , Ananth CV . Trends in spontaneous and indicated preterm delivery among singleton gestations in the United States, 2005–2012. Obstet Gynecol. 2014;124(6):1069‐1074. 25415156 10.1097/AOG.0000000000000546

[6] Aughey H , Jardine J , Knight H , et al. Iatrogenic and spontaneous preterm birth in England: a population‐based cohort study. BJOG. 2023;130(1):33‐41. 36073305 10.1111/1471-0528.17291 PMC10092353

[7] Shepherd ES , Goldsmith S , Doyle LW , et al. Magnesium sulfate before preterm birth for neuroprotection: an updated Cochrane systematic review. Obstet Gynecol. 2024;144(2):161‐170. 38830233 10.1097/AOG.0000000000005644 PMC11250087

[8] Abramson D , Reid DE . Use of relaxin in treatment of threatened premature labor. J Clin Endocrinol Metab. 1955;15(2):206‐209. 13233331 10.1210/jcem-15-2-206

[9] Crowther CA , Brown J , McKinlay CJ , Middleton P . Magnesium sulphate for preventing preterm birth in threatened preterm labour. Cochrane Database Syst Rev. 2014;2014(8):CD001060. 25126773 10.1002/14651858.CD001060.pub2 PMC10838393

[10] Hendricks CH , Cibils LA , Pose SV , Eskes TK . The pharmacologic control of excessive uterine activity with isoxsuprine. Am J Obstet Gynecol. 1961;82:1064‐1078. 14036161 10.1016/s0002-9378(16)36199-3

[11] Wilson A , Hodgetts‐Morton VA , Marson EJ , et al. Tocolytics for delaying preterm birth: a network meta‐analysis (0924). Cochrane Database Syst Rev. 2022;8(8):CD014978. 35947046 10.1002/14651858.CD014978.pub2 PMC9364967

[12] Reinebrant HE , Pileggi‐Castro C , Romero CL , et al. Cyclo‐oxygenase (COX) inhibitors for treating preterm labour. Cochrane Database Syst Rev. 2015;2015(6):CD001992. 26042617 10.1002/14651858.CD001992.pub3 PMC7068172

[13] Ulmsten U , Andersson KE , Forman A . Relaxing effects of nifedipine on the nonpregnant human uterus in vitro and in vivo. Obstet Gynecol. 1978;52(4):436‐441. 714325

[14] Ulmsten U , Andersson KE , Wingerup L . Treatment of premature labor with the calcium antagonist nifedipine. Arch Gynecol. 1980;229(1):1‐5. 7362274 10.1007/BF02109822

[15] Stelzl P , Kehl S , Oppelt P , et al. Do obstetric units adhere to the evidence‐based national guideline? A Germany‐wide survey on the current practice of initial tocolysis. Eur J Obstet Gynecol Reprod Biol. 2022;270:133‐138. 35051825 10.1016/j.ejogrb.2022.01.006

[16] Excellence NIfHaC. https://www.nice.org.uk/guidance/ng25 2015.

[17] American College of O, Gynecologists' Committee on Practice B‐O . Practice bulletin No. 171: management of preterm labor. Obstet Gynecol. 2016;128(4):e155‐e164. 27661654 10.1097/AOG.0000000000001711

[18] Dagklis T , Akolekar R , Villalain C , et al. Management of preterm labor: clinical practice guideline and recommendation by the WAPM‐world Association of Perinatal Medicine and the PMF‐perinatal medicine foundation. Eur J Obstet Gynecol Reprod Biol. 2023;291:196‐205. 37913556 10.1016/j.ejogrb.2023.10.013

[19] Itakura A , Shoji S , Shigeru A , et al. Guidelines for obstetrical practice in Japan: Japan Society of Obstetrics and Gynecology and Japan Association of Obstetricians and Gynecologists 2020 edition. J Obstet Gynaecol Res. 2023;49(1):5‐53. 10.1111/jog.15438 36251613

[20] van der Windt LI , Klumper J , Duijnhoven RG , et al. Atosiban versus placebo for threatened preterm birth (APOSTEL 8): a multicentre, randomised controlled trial. Lancet. 2025;405(10483):1004‐1013. 40049187 10.1016/S0140-6736(25)00295-8

[21] Fleckenstein A . History of calcium antagonists. Circ Res. 1983;52(2 Pt 2):I3‐I16. 6339106

[22] Abdelkader NN , Awaisu A , Elewa H , El Hajj MS . Prescribing patterns of antihypertensive medications: a systematic review of literature between 2010 and 2020. Explor Res Clin Soc Pharm. 2023;11:100315. 37635839 10.1016/j.rcsop.2023.100315 PMC10448163

[23] Umemura S , Arima H , Arima S , et al. The Japanese Society of Hypertension Guidelines for the Management of Hypertension (JSH 2019). Hypertens Res. 2019;42(9):1235‐1481. 31375757 10.1038/s41440-019-0284-9

[24] Abernethy DR , Schwartz JB . Calcium‐antagonist drugs. N Engl J Med. 1999;341(19):1447‐1457. 10547409 10.1056/NEJM199911043411907

[25] Lyell DJ , Pullen KM , Mannan J , et al. Maintenance nifedipine tocolysis compared with placebo: a randomized controlled trial. Obstet Gynecol. 2008;112(6):1221‐1226. 19037029 10.1097/AOG.0b013e31818d8386

[26] Roos C , Spaanderman ME , Schuit E , et al. Effect of maintenance tocolysis with nifedipine in threatened preterm labor on perinatal outcomes: a randomized controlled trial. JAMA. 2013;309(1):41‐47. 23280223 10.1001/jama.2012.153817

[27] Vis JY , van Baaren GJ , Wilms FF , et al. Randomized comparison of nifedipine and placebo in fibronectin‐negative women with symptoms of preterm labor and a short cervix (APOSTEL‐I trial). Am J Perinatol. 2015;32(5):451‐460. 25486290 10.1055/s-0034-1390346

[28] Songthamwat S , Na Nan C , Songthamwat M . Effectiveness of nifedipine in threatened preterm labor: a randomized trial. Int J Womens Health. 2018;10:317‐323. 29942162 10.2147/IJWH.S159062 PMC6007202

[29] Hawkins JS , Wells CE , Casey BM , McIntire DD , Leveno KJ . Nifedipine for acute tocolysis of preterm labor: a placebo‐controlled randomized trial. Obstet Gynecol. 2021;138(1):73‐78. 34259466 10.1097/AOG.0000000000004436

[30] Parry E , Roos C , Stone P , Hayward L , Mol BW , McCowan L . The NIFTY study: a multicentre randomised double‐blind placebo‐controlled trial of nifedipine maintenance tocolysis in fetal fibronectin‐positive women in threatened preterm labour. Aust N Z J Obstet Gynaecol. 2014;54(3):231‐236. 24506318 10.1111/ajo.12179

[31] Leal‐Junior CC , Amorim MMR , Souza GFA , Lima AKS , Souza ASR . Effectiveness of an oral versus sublingual loading dose of nifedipine for tocolysis. Int J Gynaecol Obstet. 2020;148(3):310‐315. 31774552 10.1002/ijgo.13067

[32] Danti L , Zonca M , Barbetti L , et al. Prophylactic oral nifedipine to reduce preterm delivery: a randomized controlled trial in women at high risk. Acta Obstet Gynecol Scand. 2014;93(8):802‐808. 24773243 10.1111/aogs.12405

[33] Simhan HN , Cartis S . Inhibition of acute preterm labor. https://www.uptodate.com/contents/inhibition‐of‐acute‐preterm‐labor?search=preterm%20labor&source=search_result&selectedTitle=2~150&usage_type=default&display_rank=2 2025.

[34] Foster TS , Hamann SR , Richards VR , Bryant PJ , Graves DA , McAllister RG . Nifedipine kinetics and bioavailability after single intravenous and oral doses in normal subjects. J Clin Pharmacol. 1983;23(4):161‐170. 6863580 10.1002/j.1552-4604.1983.tb02720.x

[35] Waller DG , Renwick AG , Gruchy BS , George CF . The first pass metabolism of nifedipine in man. Br J Clin Pharmacol. 1984;18(6):951‐954. 6529535 10.1111/j.1365-2125.1984.tb02569.x PMC1463677

[36] van de Vusse D , Mian P , Schoenmakers S , et al. Pharmacokinetics of the most commonly used antihypertensive drugs throughout pregnancy methyldopa, labetalol, and nifedipine: a systematic review. Eur J Clin Pharmacol. 2022;78(11):1763‐1776. 36104450 10.1007/s00228-022-03382-3 PMC9474278

[37] Weber‐Schoendorfer C , Hannemann D , Meister R , et al. The safety of calcium channel blockers during pregnancy: a prospective, multicenter, observational study. Reprod Toxicol. 2008;26(1):24‐30. 18585452 10.1016/j.reprotox.2008.05.065

[38] Lennestal R , Otterblad Olausson P , Kallen B . Maternal use of antihypertensive drugs in early pregnancy and delivery outcome, notably the presence of congenital heart defects in the infants. Eur J Clin Pharmacol. 2009;65(6):615‐625. 19198819 10.1007/s00228-009-0620-0

[39] Vasilakis‐Scaramozza C , Aschengrau A , Cabral HJ , Jick SS . Antihypertensive drugs and the risk of congenital anomalies. Pharmacotherapy. 2013;33(5):476‐482. 23553869 10.1002/phar.1212

[40] Russell RP . Side effects of calcium channel blockers. Hypertension. 1988;11(3 Pt 2):II42–44. 3280492 10.1161/01.hyp.11.3_pt_2.ii42

[41] Hatahira H , Abe J , Hane Y , et al. Drug‐induced gingival hyperplasia: a retrospective study using spontaneous reporting system databases. J Pharm Health Care Sci. 2017;3:19. 28729910 10.1186/s40780-017-0088-5 PMC5518137

[42] van Veen AJ , Pelinck MJ , van Pampus MG , Erwich JJ . Severe hypotension and fetal death due to tocolysis with nifedipine. BJOG. 2005;112(4):509‐510. 15777455 10.1111/j.1471-0528.2004.00480.x

[43] Papatsonis DN , Carbonne B , Dekker GA , Flenady V , King JF . Severe hypotension and fetal death due to tocolysis with nifedipine. BJOG. 2005;112(11):1582‐1583. 10.1111/j.1471-0528.2005.00752.x 16225587

[44] Luewan S , Mahathep R , Tongsong T . Hypotension in normotensive pregnant women treated with nifedipine as a tocolytic drug. Arch Gynecol Obstet. 2011;284(3):527‐530. 20844887 10.1007/s00404-010-1674-z

[45] Yamasato K , Burlingame J , Kaneshiro B . Hemodynamic effects of nifedipine tocolysis. J Obstet Gynaecol Res. 2015;41(1):17‐22. 25164435 10.1111/jog.12478

[46] Ara I , Banu H . A prospective randomised trial of nifedipine versus placebo in preterm labour. Bangladesh J Obst Gynaecol. 2008;23(2):61‐64.

[47] Nijman TA , van Vliet EO , Naaktgeboren CA , et al. Nifedipine versus placebo in the treatment of preterm prelabor rupture of membranes: a randomized controlled trial: assessment of perinatal outcome by use of tocolysis in early labor‐APOSTEL IV trial. Eur J Obstet Gynecol Reprod Biol. 2016;205:79‐84. 27567363 10.1016/j.ejogrb.2016.08.024

[48] van Vliet E , Seinen L , Roos C , et al. Maintenance tocolysis with nifedipine in threatened preterm labour: 2‐year follow up of the offspring in the APOSTEL II trial. BJOG. 2016;123(7):1107‐1114. 26330379 10.1111/1471-0528.13586

[49] Zamani M , Alimi R , Arabi SM , Moradi M , Azmoude E . Comparison of the efficacy of nifedipine with ritodrine, nitroglycerine and magnesium sulfate for the management of preterm labor: a systematic review and meta‐analysis. BMC Pregnancy Childbirth. 2024;24(1):318. 38664622 10.1186/s12884-024-06497-w PMC11044545

[50] Bell EF , Hintz SR , Hansen NI , et al. Mortality, in‐hospital morbidity, care practices, and 2‐year outcomes for extremely preterm infants in the US, 2013–2018. JAMA. 2022;327(3):248‐263. 35040888 10.1001/jama.2021.23580 PMC8767441

